# Intersections between polyvictimisation and mental health among adolescents in five urban disadvantaged settings: the role of gender

**DOI:** 10.1186/s12889-017-4348-y

**Published:** 2017-07-04

**Authors:** Mphatso Kamndaya, Pedro T. Pisa, Matthew F. Chersich, Michele R. Decker, Adesola Olumide, Rajib Acharya, Yan Cheng, Heena Brahmbhatt, Sinead Delany-Moretlwe

**Affiliations:** 1 Wits RHI, Faculty of Health Sciences, University of the Witwatersrand, Johannesburg, South Africa; 2 Africa Centre for Migration and Society, University of the Witwatersrand, Johannesburg, South Africa; 3 Department of Population, Family and Reproductive Health, Johns Hopkins Bloomberg School of Public Health, Baltimore, MD USA; 4 Institute of Child Health, College of Medicine, University of Ibadan/University College Hospital Ibadan, Ibadan, Nigeria; 5 Population Council, New Delhi, India; 6 Shanghai Institute of Planned Parenthood Research, Shanghai, China; 7 Family Planning NSW, Sydney, Australia

**Keywords:** Polyvictimisation, Mental health, Adolescents, Gender differences, Urban disadvantaged environments

## Abstract

**Background:**

Polyvictimisation (PV) – exposure to violence across multiple contexts – causes considerable morbidity and mortality among adolescents. Despite high levels of violence in urban disadvantaged settings, gender differences in associations between PV and mental health have not been well established.

**Methods:**

We analysed data from a survey with 2393 adolescents aged 15-19 years, recruited using respondent-driven sampling from urban disadvantaged settings in Baltimore (USA), Delhi (India), Ibadan (Nigeria), Johannesburg (South Africa) and Shanghai (China). PV was defined as exposure to two or more types of violence in the past 12 months with family, peers, in the community, or from intimate partners and non-partner sexual violence. Weighted logistic regression models are presented by gender to evaluate whether PV is associated with posttraumatic stress, depression, suicidal thoughts and perceived health status.

**Results:**

PV was extremely common overall, but ranged widely, from 74.5% of boys and 82.0% of girls in Johannesburg, to 25.8 and 23.9% respectively in Shanghai. Community violence was the predominant violence type, affecting 72.8–93.7% across the sites. More than half of girls (53.7%) and 45.9% of boys had at least one adverse mental health outcome. Compared to those that did not report violence, boys exposed to PV had 11.4 higher odds of having a negative perception of health (95%CI adjusted OR = 2.45-53.2), whilst this figure was 2.58 times in girls (95%CI = 1.62-4.12). Among girls, PV was associated with suicidal thoughts (adjusted OR = 4.68; 95%CI = 2.29-9.54), posttraumatic stress (aOR = 4.53; 95%CI = 2.44-8.41) and depression (aOR = 2.65; 95%CI = 1.25-5.63). Among boys, an association was only detected between PV and depression (aOR = 1.82; 95%CI = 1.00-3.33).

**Conclusion:**

The findings demonstrate that PV is common among both sexes in urban disadvantaged settings across the world, and that it is associated with poor mental health outcomes in girls, and with poor health status in both girls and boys. Clearly, prevention interventions are failing to address violence exposure across multiple contexts, but especially within community settings and in Johannesburg. Interventions are needed to identify adolescents exposed to PV and link them to care, with services targeting a range of mental health conditions among girls and perhaps focusing on depression among boys.

**Electronic supplementary material:**

The online version of this article (doi:10.1186/s12889-017-4348-y) contains supplementary material, which is available to authorized users.

## Background

Polyvictimisation (PV), or exposure to violence across multiple contexts, causes significant short- and long-term morbidity and even mortality among adolescents [1–3]. Research interest has grown in understanding the impact of multiple exposures to violence across a range of contexts including within the family and community, and from intimate partners and peers [4, 5]. Globally, rates of exposure to PV in adolescents vary considerably. In high-income countries, for example, estimates of PV range from 10 to 93% [5–10]. Rates of PV are generally higher in low- and middle-income countries (LMICs) [11], reaching almost 95% in one community-based sample in Cape Town, South Africa, for example [12].

A constellation of social vulnerabilities means that adolescents living in disadvantaged urban settings are at a particularly high risk for PV [12–15], and there are often very few social and health resources to counter these vulnerabilities [15, 16]. Victims of PV are at a considerably higher risk for poor mental health, as well as emotional, behavioural and developmental problems compared with those exposed to violence in a single context [17–19]. It has, however, not been clearly established whether the impact of PV on mental health varies by gender in these settings. The burden of PV and the associated mental health outcomes might be more profound for girls than boys, given high levels of sexual violence against females [4, 20]. Previously, we showed that adolescent women living in disadvantaged environments experienced a significant burden of gender-based violence and associated health consequences [15]. Equally however, boys may be impacted more by male-on-male physical violence. Studies that have explored differences in prevalence of PV by gender have inconsistent findings [4, 20], suggesting that variations in forms or combinations of violence may create different risk profiles, and that these may be further moderated by age, place of residence and a host of other factors. Multi-site studies, applying standardized survey and analytical methodology, can explore gender differences in detail, and overcome the present problems with generalising findings from studies of single populations. Conclusions that are generalizable are needed to guide broad recommendations for victimisation prevention and care initiatives in disadvantaged urban settings. These, in turn, can then be adapted for implementation in similar settings.

Against this backdrop, we have a unique opportunity to extend the literature by examining gender differences in the association between PV and mental health among adolescents in five economically-distressed urban settings, across three continents. Also, the prevalence of PV, each type of violence and mental health problems in the whole study population are described. This provides useful insights into how adolescents’ lived experiences vary in disadvantaged, urban settings across the world.

## Methods

### Sample and procedures

Data were drawn from the cross-sectional Well Being of Adolescents in Vulnerable Environments (WAVE) study, which enrolled adolescents aged 15-19 years living in disadvantaged urban settings in Baltimore (USA), Johannesburg (South Africa), Ibadan (Nigeria), Delhi (India) and Shanghai (China) in 2013. In Shanghai, recruitment was limited to migrant adolescents. Respondent-driven sampling (RDS) was used to recruit approximately 500 participants in each site. All interviews were conducted using computer-assisted self-interview instruments.

Full details of the study methodology [21], measures of violence [15], indicators of mental health and their validity [22], and of the study population [23] are presented in previous publications. All adolescents gave written informed consent/assent. In addition, parents or guardians of adolescents aged 15–17 years gave written informed consent.

### Study measures

The study measures drew on existing validated tools wherever possible. Where such tools could not be located, we developed the tools during a formative study phase that preceded the survey [24]. *Polyvictimisation* was defined as exposure to two or more types of victimisation in the past 12 months, which consisted of: *family violence* encompassing the experience or witnessing of at least one act of physical or psychological violence perpetrated by a family member; *peer violence* including the experience or witnessing of at least one act of physical or psychological violence perpetrated by peers of either sex; *community violence,* entailing the witnessing of criminal activities, such as drug deals, interpersonal violence with or without a weapon, and arrests, or the experience of having one’s house broken into (these measures were drawn from National Survey of American life) [25, 26]; and *intimate partner violence* (IPV), which included both physical and sexual violence; and *non-partner sexual violence* (NPSV), (i.e., that perpetrated by someone who was not a dating partner or spouse) [15]. IPV, separated into sexual and physical IPV, was assessed only among girls given that experiencing violence from a partner is more common and more severe among women [27]. Given that male IPV was not assessed, boys had a maximum of four types of violence, while girls had a maximum of six. Respondents who had never had an intimate partner were classified as not having experienced IPV.

### Mental health outcomes

Mental health outcomes were identified through self-reported responses to scales assessing symptoms of: *posttraumatic stress* (Posttraumatic stress disorder checklist-civilian version used for diagnosing posttraumatic stress) [28]; *depression* (the short 10-item version of the Centre for Epidemiological Studies Depression Scale) [29]; and *suicidal thoughts* in the past 12 months [18]. Ever having *planned or attempted suicide* was also reported. We also describe the prevalence of: heavy alcohol use (ever having consumed five or more alcohol drinks within 2 h, or being drunk at least once a month [4]); a negative perception of overall health status (responded ‘fair’ or ‘poor’ to health status measure); and having sought treatment for mental health problems in the past 12 months.

### Data analysis

Weights were generated via the RDS II estimator [30] to account for inter-cluster correlations. Given differences in the age distribution across centres, a post-stratification age weight was also developed and combined with the RDS weight. Weighted means and percentages were computed for continuous and categorical variables respectively, and compared by gender within each site.

Logistic regression models using weighted data were fitted to evaluate the relationship between PV and perceived health status, posttraumatic stress, depression and suicidal thoughts, separately for males and females. Multivariate logistic regression models were then fitted, again separately by gender, for two-tailed tests of the null hypothesis that odds ratio (OR) = 1, to evaluate the relationship between between PV and each of the four outcome variables, adjusted for age and city of residence. The reference group in these models for PV was those with no experience of violence and for the city variable, Baltimore. Adjusted ORs (aOR) and their corresponding 95% confidence intervals were calculated for each study outcome. All analyses used STATA v.12 statistical software.

## Results

### Prevalence of past-year polyvictimisation, by gender within each city

Past-year PV was extremely common overall, but ranged from a high of 74.5% of boys and 82.0% of girls in Johannesburg, to a low of 25.8 and 23.9% respectively in Shanghai ((Table 1). Though a considerable portion of girls had experienced PV in Delhi (60.5%) and Ibadan (50.2%), levels among boys in these settings were even higher (71.0 and 62.5% respectively). By contrast, levels of PV were notably higher among girls than boys in Baltimore (71.1% versus 42.5%), mostly due to high levels of physical IPV experienced by girls (Table 1).

**Table 1:** Summary measures of family, community, peer and intimate partner, and non-partner sexual violence, and number of types of victimisation, by gender within each city

	Baltimore W%, n			Delhi W%, n			Ibadan W%, n			Johannesburg W%, n			Shanghai W%, n		
Category of violence	Male *N* = 276	Female *N* = 195	*P*	Male *N* = 250	Female *N* = 250	*P*	Male *N* = 233	Female *N* = 232	*P*	Male *N* = 273	Female *N* = 224	*P*	Male *N* = 235	Female *N* = 220	*P*
Family violence	16.3, 50	24.0, 52	0.02	34.8, 84	52.4, 111	0.003	52.2, 117	44.8, 97	0.24	44.5, 118	41.4, 87	0.53	20.3, 48	25.1, 51	0.17
Community violence	86.4, 219	78.8, 165	0.14	84.6, 207	75.8, 193	0.04	77.0, 161	72.8, 158	0.23	93.7, 255	88.4, 199	<0.001	29.8, 85	22.6, 52	0.03
Peer violence	39.6, 104	37.2, 72	0.40	77.3, 179	46.9, 96	0.01	72.4, 59	47.5, 42	0.23	67.6, 130	63.9, 133	0.66	61.6, 40	44.3, 33	0.02
Physical IPV	–	25.1, 48	–	–	15.9, 7	–	–	25.6, 13	–	–	30.6, 65	–	–	7.9, 9	–
Sexual IPV	–	9.9, 19	–	–	7.4, 5	–	–	15.4, 11	–	–	19.5, 44	–	–	1.7, 4	–
Non-partner SV	15.1, 42	48.7, 112	<0.001	16.7, 42	33.4, 85	0.04	50.0, 97	26.7, 53	<0.001	34.1, 98	49.4, 117	0.006	16.4, 43	34.3, 66	0.001
Number of types of victimisation^a^															
0	11.8, 33	10.7, 8	0.01	6.5, 17	12.4, 30	0.05	10.8, 39	17.5, 45	0.50	2.5, 6	6.0, 10	0.15	43.9, 93	38.8, 94	0.50
1	46.5, 125	29.8, 47	<0.001	24.4, 55	27.4, 68	0.18	22.4, 48	31.3, 70	0.04	22.9, 63	12.7, 30	0.04	31.1, 81	35.1, 73	0.77
2 or more (PV)	42.5, 118	71.1, 139	0.01	71.0, 178	60.5, 152	0.04	62.5, 146	50.2, 117	0.04	74.5, 204	82.0, 184	0.04	25.8, 61	23.9, 53	0.51

*W%* weighted percentage, *n* number of cases, *N* sample size

^a^Sum of numbers of experiences of family violence, community violence, peer violence, physical IPV, sexual IPV and non-partner sexual violence

When examining the relative frequency of different types of victimisation, the most common was community violence in all cities (range 72.8–93.7%), except in Shanghai, where peer violence predominated (61.6% in boys and 44.3% in girls; Table 1). Experiences of community violence were higher for adolescent boys than girls in Delhi, Johannesburg and Shanghai. No differences in experience of family violence were observed between adolescent girls and boys in Ibadan, Johannesburg, and Shanghai, while in Delhi and Baltimore, girls reported considerably higher levels of family violence than boys, and as compared with girls elsewhere. A remarkable 40.7% of girls in Delhi had been physically hurt in their home and 29.6% had been hurt in a fight with peers (Additional file 1). In Baltimore, girls reported that family violence frequently took the form of being pushed, grabbed, shoved or threatened in their homes, which occurred almost twice as frequently than in boys.

Peer violence was experienced more frequently by adolescent girls in Delhi and Shanghai, compared to boys in those cities (Additional file 2). All forms of sexual IPV were highest among girls in Johannesburg, as was overall occurrence of physical IPV, with almost a third reporting having been slapped and a quarter pushed by an intimate partner. Levels of NPSV were several fold higher in girls than boys in Baltimore, Delhi and Shanghai, while a considerable portion of boys in Johannesburg had experienced this form of violence (34.1% versus 49.4% in girls) and it was even more common in boys than girls in Ibadan (50.0% versus 26.7% in girls; Additional file 3).

### Prevalence of mental health outcomes, by gender within each city

Across all sites, posttraumatic stress was considerably more common in adolescent girls than boys, aside for Ibadan where 27.9% of boys and 22.2% of girls had posttraumatic stress. Similarly, a higher proportion of adolescent girls than boys reported depressive symptoms in Shanghai (33.5%), Ibadan (40.2%) and Johannesburg (46.1%), which had the highest levels of depression for both sexes. As an exception, boys had higher levels of depression than girls in Baltimore (32.4% versus 27.3%). There were also gender differences in suicidal thoughts, plans and attempts across the sites, with a female predominance in Baltimore, Johannesburg and Shanghai. Most especially in Shanghai, the prevalence of suicidal thoughts, plans and attempts was several fold higher among girls than boys. The most consistent differences in mental health outcomes between adolescent boys and girls were observed in Shanghai and Johannesburg, where a higher proportion of girls than boys had each of the mental health outcomes measured.

With regard to co-morbid conditions, heavy alcohol use (assessed among current alcohol users only) was uncommon in Delhi, was considerably more frequent in boys than girls in Ibadan and Johannesburg, and occurred roughly in the same proportion of both sexes in Baltimore and Shanghai (41 to 48%). Negative perceptions of overall health status were notably more common in girls than boys in Baltimore (10.1 versus 4.1%) and Shanghai (16.8% against 5.2%). Treatment seeking for mental health concerns was infrequent (around 1% overall), irrespective of setting (Table 2).

**Table 2:** Prevalence of mental health indicators and other outcome measures, by gender within each city

Mental health and other outcome measures	Baltimore W%, n			Delhi W%, n			Ibadan W%, n			Johannesburg W%, n			Shanghai W%, n		
	Male *N* = 276	Female *N* = 195	*P*	Male *N* = 250	Female *N* = 250	*P*	Male *N* = 233	Female *N* = 232	*P*	Male *N* = 273	Female *N* = 224	*P*	Male *N* = 235	Female *N* = 220	*P*
Poor or fair health	4.1, 11	10.1, 18	<0.001	9.7, 26	15.4, 38	0.31	4.4, 10	3.2, 8	0.16	13.8, 36	11.0, 23	0.30	5.2, 16	16.8, 29	0.08
Posttraumatic stress															
Scores ≥13^a^	26.5,71	30.6,65	0.46	17.4,30	27.4,75	0.01	27.9,55	22.2,53	0.34	47.6131	58.5134	0.15	22.2,50	30.5,61	<0.001
Depression															
Scores ≥11^b^	32.4,79	27.3,60	0.06	20.7, 49	14.5, 46	0.21	28.9, 56	40.2, 73	<0.001	37.2106	46.1, 94	0.04	27.1,50	33.5, 74	0.01
Suicide															
Suicidal thoughts	16.0,46	26.9,45	0.04	14.0,33	8.0,22	0.08	23.8,43	19.0,47	0.63	32.7,77	39.3,86	0.19	19.4,29	37.6,63	<0.001
Plans	6.4,24	13.9, 26	0.09	4.0,9	2.7,6	0.11	18.4,34	14.2,34	0.54	19.5,40	24.6,49	0.33	6.0,8	11.2,21	0.01
Attempts	4.8,23	7.4,14	0.07	4.1,8	1.8,5	0.12	18.9,31	14.1,33	0.59	11.9,26	10.1,29	0.20	2.5, 3	6.0,11	<0.001
Alcohol use	*N* = 70	*N* = 52		*N* = 9	*N* = 1		*N* = 36	*N* = 18		*N* = 169	*N* = 87		*N* = 142	*N* = 97	
Heavy alcohol use	48.2,35	41.1,20	0.54	4.9, 1	0.0, 0	0.83	61.0, 16	10.8, 2	0.01	55.6,91	38.0, 38	0.04	42.1,67	42.3,37	0.99
Sought mental health services in past 12 months	1.2, 4	2.8,7	0.01	1.6, 3	1.4, 4	0.04	0.8, 1	0.0, 0	0.14	0.2, 1	0.3, 2	0.04	1.8, 3	0.6, 2	0.01

*W%* weighted percentage, *n* number of cases, *N* sample size

^a^Posttraumatic stress disorder checklist-civilian version used for diagnosing posttraumatic stress

^b^The short 10-item version of the Center for Epidemiological Studies Depression Scale. Heavy alcohol use assessed among current drinkers

### Polyvictimisation and its association with health status and mental health

The first part of this section presents the findings of the four mental health outcomes as a function of PV, separately for males and females (Tables 3 and 4). The second part sums the associations noted between these outcomes, and age and city (Tables 3 and 4).

**Table 3:** Unadjusted odds ratios (with 95% CI) for associations between number of types of victimisation and mental health by gender

	Poor or fair health status				Posttraumatic stress			
Independent variables	Male (*n* = 1208)		Female (*n* = 1099)		Male (*n* = 1121)		Female (*n* = 1069)	
Number of types of victimisation	OR (95%CI)	*P*	OR (95%CI)	*P*	OR (95%CI)	*P*	OR (95%CI)	*P*
0 (Ref)	1.00		1.00		1.00		1.00	
1	**7.99 (2.05, 31.25)**	0.004	1.11 (0 .46, 2.67)	0.811	1.63 (0.86, 3.11)	0.129	1.26 (0.68, 2.34)	0.460
2 or more (PV)	**12.84 (4.21, 9.20)**	<0.001	1.82 (0.80, 4.14)	0.149	**2.29 (1.02, 5.17)**	0.046	**4.39 (2.13, 9.04)**	<0.001
R2 value	0.02		0.02		0.02		0.06	
	Depression				Suicidal thoughts			
	Male (*n* = 1151)		Female (*n* = 1043)		Male (*n* = 1211)		Female (*n* = 1102)	
Number of types of victimisation	OR (95%CI)	*P*	OR (95%CI)	*P*	OR (95% CI)	*P*	OR (95% CI)	*P*
0 (Ref)	1.00		1.00		1.00		1.00	
1	1.22 (0.48, 3.08)	0.661	0.83 (0.58, 1.19)	0.304	0.83 (0.38, 1.77)	0.612	1.79 (0.82, 3.92)	0.139
2 or more (PV)	1.74 (0.94, 3.19)	0.074	**2.12 (1.01, 4.46)**	0.049	1.72 (0.91, 3.25)	0.090	**2.90 (1.06, 7.93)**	0.039
R2 value	0.02		0.02		0.02		0.04	

Bold OR values indicate significance at *p* < 0.05. 95%CI = 95% confidence interval

**Table 4:** Odds ratios of associations between polyvictimisation and mental health, by gender, adjusted for age and city of residence

Independent variables	Poor or fair health status				Posttraumatic stress			
	Male (*n* = 1208)		Female (*n* = 1099)		Male (*n* = 1121)		Female (*n* = 1069)	
Number of types of victimisation	aOR (95%CI)	*P*	aOR (95%CI)	*P*	aOR (95%CI)	*P*	aOR (95%CI)	*P*
0 (Ref)	1.00		1.00		1.00		1.00	
1	**7.90 (1.43, 43.56)**	0.019	**1.35 (0.65, 2.82)**	0.415	1.33 (0.73, 2.42)	0.341	1.41 (0.78, 2.54)	0.248
2 or more (PV)	**11.43 (2.45,53.21)**	0.003	**2.58 (1.62, 4.12)**	<0.001	1.79 (0.83, 3.88)	0.134	**4.53 (2.44, 8.41)**	<0.001
Age (years)	1.05 (0.85, 1.30)	0.635	0.95 (0.85,1.06)	0.325	**1.20 (1.03, 1.40**)	0.024	0.95 (0.84, 1.09)	0.454
City of residence								
Baltimore (Ref)	1.00		1.00		1.00		1.00	
Delhi	2.31 (0.77, 6.88)	0.128	1.61 (0.85, 3.09)	0.142	**0.54 (0.38, 0.78)**	0.002	0.83 (0.60, 1.15)	0.256
Ibadan	0.99 (0.48, 2.08)	0.988	**0.31 (0.13, 0.71)**	0.007	0.99 (0.66, 1.51)	0.979	**0.69 (0.48, 0.99)**	0.046
Johannesburg	**3.22 (1.63, 6.36)**	0.001	0.96 (0.55, 1.68)	0.876	**2.38 (1.50, 3.77)**	0.001	**2.66 (1.53, 4.62)**	0.001
Shanghai	2.04 (0.67, 6.20)	0.198	**2.45 (1.33, 4.52)**	0.005	0.91 (0.64, 1.30)	0.596	**1.68 (1.20, 2.34)**	0.003
R2 value	0.05		0.06		0.07		0.09	
	Depression				Suicidal thoughts			
	Male (*n* = 1151)		Female (*n* = 1043)		Male (*n* = 1211)		Female (*n* = 1102)	
Number of types of victimisation	aOR (95%CI)	*P*	aOR (95%CI)	*P*	aOR (95% CI)	*P*	aOR (95% CI)	*P*
0 (Ref)	1.00		1.00		1.00		1.00	
1	1.15 (0.48, 2.77)	0.747	0.96 (0.65, 1.40)	0.819	0.85 (0.41, 1.74)	0.640	**2.46 (1.17, 5.18)**	0.019
2 or more (PV)	**1.82 (1.00, 3.33)**	0.050	**2.65 (1.25, 5.63)**	0.013	1.65 (0.88, 3.11)	0.116	**4.68 (2.29, 9.54)**	<0.001
Age (years)	**1.19 (1.01, 1.39)**	0.034	1.05 (0.85, 1.29)	0.641	0.93 (0.82, 1.05)	0.220	0.98 (0.84, 1.15)	0.831
City of residence								
Baltimore (Ref)	1.00		1.00		1.00		1.00	
Delhi	**0.49 (0.31, 0.76)**	0.002	**0.44 (0.34, 0.55)**	<0.001	0.68 (0.40, 1.16)	0.151	**0.22 (0.19, 0.27)**	<0.001
Ibadan	**0.73 (0.57, 0.95)**	0.019	**1.97 (1.49, 2.61)**	<0.001	1.37 (0.84, 2.23)	0.193	0.68 (0.40, 1.15)	0.142
Johannesburg	1.09 (0.80, 1.50)	0.571	**1.94 (1.50, 2.52)**	<0.001	**2.00 (1.34, 2.99)**	0.001	**1.49 (1.04, 2.14)**	0.030
Shanghai	0.88 (0.69, 1.12)	0.276	**1.94 (1.39, 2.70)**	<0.001	**1.30 (1.02, 1.66)**	0.033	**2.62 (1.91, 3.59)**	<0.001
R2 value	0.04		0.05		0.04		0.10	

Bold OR values indicate significance at *p* < 0.05

*aOR* adjusted odds ratios, *95%CI* 95% confidence interval

For each outcome, the ORs increased for both genders as the number of types of victimisation increased (Tables 3 and 4). There were, however, differences in the magnitude of increase, and statistical significance, according to gender. Moreover, considerable differences were noted between effect sizes in unadjusted and adjusted models.

PV was strongly associated with negative perceptions of overall health for both boys and girls. Specifically, boys that experienced PV had 11.43 higher odds of having a negative perception of health than those who had never experienced violence (95%CI: 2.45-53.21), whereas these odds were 2.58 in girls with PV (95%CI: 1.62-4.12; Table 4).

Gender differences were also observed in the association between PV and the mental health outcome variables assessed. Independent of age and city of residence, girls that experienced PV had an increased odds of suicidal thoughts (aOR = 4.68; 95%CI: 2.29-9.54), posttraumatic stress (aOR = 4.53; 95%CI: 2.44-8.41) and depression (aOR = 2.65; 95%CI: 1.25-5.63), while the sizes of point estimates for these three associations were lower among boys. Depression was the only mental health outcome associated with PV in boys (aOR = 1.82; 95%CI = 1.00-3.33).

Several other findings in the multivariate analysis bear mention. Firstly, associations were detected between having one episode of victimisation and negative perceptions of health for boys (aOR = 7.90; 95%CI: 1.43-43.56) and suicidal thoughts for girls (aOR = 2.46; 95%CI: 1.17-5.18) compared to those who had never experienced violence. None of the estimated associations between having one episode of victimisation and the other outcomes were statistically significant, with the point estimates ranging from 0.85-1.41).

Secondly, city of residence was an important determinant of all outcomes assessed, independent of age and of having experienced victimisation. Negative perceptions of health were more common among boys in Johannesburg (aOR = 3.22; 95%CI: 1.63-6.36) and girls in Shanghai (aOR = 2.45; 95%CI: 1.33-4.52), than in Baltimore, but less frequent in girls living in Ibadan (aOR = 0.31; 95%CI: 0.13-0.71). Most strikingly, however, being in Johannesburg, as opposed to in Baltimore, was associated with around a two-fold higher odds of all the mental health outcomes, aside from depression in boys. The converse was true of Delhi, where point estimates were the lowest for both genders.

Compared with Baltimore, girls in Ibadan had less posttraumatic stress, but more frequently had depression. Of note, the girls in Shanghai had 2.6 fold higher odds of suicidal thoughts than those in Baltimore (95%CI: 1.91-3.59), and also had higher odds of posttraumatic stress and depression. In Shanghai, aside from a 1.3 fold higher odds of suicidal thoughts in boys than in Baltimore, no significant differences were detected between boys in these two cities. Finally, age was associated with posttraumatic stress and depression for boys, where ORs of the condition rose with age.

## Discussion

We primarily aimed to explore whether there are gender differences in the association between PV and mental health outcomes among urban disadvantaged adolescents in five cities. In this regard, we demonstrate that whilst past-year PV is remarkably common in adolescents in all five cities, affecting around three quarters of adolescents in four of the five cities, its effects on mental and physical health vary, and differ by gender. PV was strongly associated with negative perceptions of overall health for both boys and girls, but the effect sizes tended to be higher for boys than girls. By contrast, girls that experienced PV had an increased likelihood of suicidal thoughts, posttraumatic stress and depression, while for boys these associations were not significant in adjusted analyses. The point estimates of associations between PV and the mental health outcomes were lower among the boys and not significant aside from depression. We also illustrated clearly that city of residence is an important determinant of all associations studied, even independent of whether boys or girls experienced victimisation.

Most previous studies involved data from one site only and report on single types of victimisation, making it difficult to compare our results to those of others. Where differences are observed between studies, these may also be accounted for by variations in the definition of exposure and outcomes. Nevertheless, our findings are broadly consistent with that of an Italian study among university students, which concluded that PV was associated with a high risk of depression, panic attacks, heavy alcohol use, eating problems, suicidal ideation and attempts, and a negative perception of overall health [4]. Other studies in high-income countries [20] and LMICs [17] have also found that adolescent girls who experienced violence were at a higher risk for depression than their male counterparts.

Somewhat unexpectedly, in quite a few instances, boys carried an equal burden of violence and mental illness as girls. This reinforces the importance of overcoming the challenges of reaching men with health services, starting with adolescent boys. Notably, rates of some outcomes among boys escalate rapidly with age.

Addressing the burden of mental illness in adolescents would require site-specific, innovative interventions that are able to take account of the diverse forms of gendered behaviours in different settings. This seems unlikely to occur in the near future, as access to mental health services is dismal for each of the conditions measured and in all sites. With a few exceptions, none of the affected adolescents, whether male or female, are currently receiving treatment. Even if services were available, stigma, especially prevalent among boys, would remain a major obstacle to treatment [31]. Stigma leads to secrecy and avoidance of treatment, and initiatives to counter stigma have met with little success to date [32]. Moreover, as noted in a systematic review, adolescents across the world commonly hold uninformed, negative views of mental health services and professionals, and may believe that accessing such services is a ‘sign of weakness’ [33]. However, adolescents, especially adolescent boys are proficient in the use of mobile technologies and this has the potential to improve uptake and accessibility of mental health interventions [34].

Regarding city of residence, boys and girls in Johannesburg had higher ORs than Baltimore for almost all indices assessed. In other words, something about living in Johannesburg – other than PV, gender and being disadvantaged – is responsible for the poor mental and physical health in this city. The high levels of PV in Johannesburg is perhaps not surprising given that South Africa is one of the most violent countries in the world [35]. Living in Delhi, by contrast, appears to be protective against PV and mental illness, though girls in Delhi reported alarming levels of physical violence in the home and from peers. Equally, physical violence from intimate partners affected a quarter to a third of girls in Baltimore, Ibadan and Johannesburg. Variations in violence by gender might partly be explained by differences in gender inequality across sites. Based on the UNDP Gender Inequality Index [36], inequality is highest in Delhi of the cities included in the study, potentially explaining the high levels of family violence among girls in the city. Gender inequalities are also high in Johannesburg, again correlating with high rates of violence among girls and boys in that area. By contrast, in Shanghai, which has the lowest levels of victimisation in the study, inequalities are lowest of the five cities according to the UNDP Index.

Several limitations and strengths of this study should be noted. The cross-sectional design limits the ability to draw causal inferences about the associations detected. We also lack data on the intensity of the individual elements of violence assessed, which implies that severe repeated family violence, for example, would still be counted only as one form of violence. The relatively large samples drawn from five cities across three continents are a strength of the study and increase the validity of the study’s assertions. Longitudinal studies could more fully unravel the immediate, underlying and environmental factors that account for PV and its impacts, and identify different patterns of PV and its effects over the life course. Additionally, qualitative analyses could deepen our understanding of how victimisation is experienced by adolescents in different settings [37], and how they develop coping strategies in response.

## Conclusions

In conclusion, this study sheds some light on the role of gender in the intersections between PV and mental health among adolescents in five urban disadvantaged settings. The results show that PV is associated with the mental health outcomes for girls, and poor self-rated health and depression for the boys. Girls who have experienced PV require specific targeting with services that address posttraumatic stress and depression. Overall, the findings highlight the dire need for interventions that reduce violence exposure and improve safety in these settings. For instance, there is evidence from Uganda, which shows the potential to reduce violence by focussing on social mobilisation [38]. It is also possible to modify violence outcomes within programmatic time frames when interventions are delivered at a structural level [39]. mHealth interventions for adolescents show much promise, such as the rapidly expanding Circle of Six smartphone application which addresses gender-based violence [27]. Historically, intervention systems address specific forms of violence in general - for example, IPV support, family violence support etc., but rarely consider the overlap between these [16].

This study highlights the need to shift this approach to one which understands and addresses the interacting compounding impacts of violence exposure across multiple contexts. Moreover, high levels of PV indicate that current preventive efforts are poorly, if at all, effective, and the effects of this are magnified by the absence of treatment for the mental health consequences thereof. This highlights the critical importance of identifying new funding for interventions to prevent victimisation across multiple contexts and to mitigate its short- and long-term consequences among adolescents of both sexes in these settings.

## Additional files

Additional file 1: Table S1. Prevalence of family and community violence, by gender within each city. (DOCX 36 kb)

Additional file 2: Table S2. Prevalence of peer violence, by gender within each city. (DOCX 32 kb)

Additional file 3: Table S3. Prevalence of intimate partner and non-partner sexual violence, by gender within each city. (DOCX 35 kb)

## Abbreviations

IPV: Intimate partner violence
LMIC: Low- and Middle-Income Countries
NPSV: Non-partner sexual violence
PV: Polyvictimisation
RDS: Respondent-driven Sampling
UNDP: United Nations Development Programme
WAVE: Wellbeing of Adolescents in Vulnerable Environments Study
