# Supplementary material for: Intersections between polyvictimisation and mental health among adolescents in five urban disadvantaged settings: the role of gender
Source: BMC Public Health. 2017 Jul 4;17(Suppl 3):41–50. doi: 10.1186/s12889-017-4348-y (PMC5498854; doi:10.1186/s12889-017-4348-y)
Supplement: Supplementary file 1 — Prevalence of family and community violence, by gender within each city. (DOCX 36 kb) [file 12889_2017_4348_MOESM1_ESM.docx]

Table S1: Prevalence of family and community violence by gender within each city

|  | **Baltimore**  **W%, n** | | | **Delhi**  **W%, n** | | | **Ibadan**  **W%, n** | | | **Johannesburg**  **W%, n** | | | **Shanghai**  **W%, n** | | |
| --- | --- | --- | --- | --- | --- | --- | --- | --- | --- | --- | --- | --- | --- | --- | --- |
| **Category of violence** | **Male**  **N = 276** | **Female**  **N = 195** | ***P*** | **Male**  **N = 250** | **Female**  **N = 250** | ***P*** | **Male**  **N = 233** | **Female**  **N = 232** | ***P*** | **Male**  **N = 273** | **Female**  **N = 224** | ***P*** | **Male**  **N = 235** | **Female**  **N = 220** | ***P*** |
| **Family violence** | 16.3, 50 | 24.0, 52 | **0.02** | 34.8, 84 | 52.4, 111 | **0.003** | 52.2, 117 | 44.8, 97 | 0.24 | 44.5, 118 | 41.4, 87 | 0.53 | 20.3, 48 | 25.1, 51 | 0.17 |
| Witness a gun or other weapon in home | 1.6, 3 | 3.5, 5 | 0.50 | 0.4, 1 | 1.5, 2 | **0.001** | 15.8, 36 | 7.8, 20 | 0.06 | 6.2, 22 | 3.6, 9 | **0.001** | 0.7, 3 | 1.3, 4 | 0.27 |
| Pushed, grabbed or shoved by someone in home | 11.9, 35 | 18.7, 39 | **0.003** | 20.1, 49 | 15.7, 38 | 0.24 | 29.9, 71 | 23.8, 50 | 0.48 | 18.2, 54 | 22.7, 51 | 0.18 | 17.3, 37 | 17.5, 39 | 0.94 |
| Threatened verbally in home | 7.5, 25 | 13.8, 29 | **0.01** | 16.8, 39 | 11.9, 26 | 0.28 | 31.6, 67 | 21.5, 47 | 0.18 | 28.1, 75 | 24.9, 50 | 0.45 | 4.0, 11 | 13.9, 19 | **0.01** |
| Threatened with a gun, knife or other weapon in home | 2.4, 6 | 3.7, 8 | 0.51 | 2.0, 3 | 1.5, 2 | 0.80 | 14.4, 30 | 13.9, 29 | 0.83 | 15.5, 36 | 10.4, 19 | 0.35 | 1.7, 6 | 2.5, 3 | 0.19 |
| Hurt with a gun, knife or other weapon in home | 3.9, 18 | 10.1, 23 | **0.001** | 18.3, 51 | 40.7, 87 | **<0.001** | 21.8, 46 | 21.8, 49 | 0.99 | 20.0, 51 | 22.6, 52 | 0.64 | 3.8, 9 | 5.2, 10 | 0.26 |
| **Community violence** | 86.4, 219 | 78.8, 165 | 0.14 | 84.6, 207 | 75.8, 193 | **0.04** | 77.0, 161 | 72.8, 158 | 0.23 | 93.7, 255 | 88.4, 199 | **<0.001** | 29.8, 85 | 22.6, 52 | **0.03** |
| Hear guns being shot | 62.9, 170 | 60.0, 133 | 0.51 | 5.3, 18 | 5.3, 14 | 0.97 | 44.1, 100 | 36.9, 82 | 0.27 | 69.8, 198 | 62.7, 146 | **0.02** | 1.7, 6 | 0.0, 0 | **0.01** |
| Witnessed somebody get arrested | 73.1, 198 | 74.0, 152 | 0.83 | 61.5, 156 | 47.6, 123 | 0.08 | 53.2, 117 | 50.4, 113 | 0.57 | 82.0, 223 | 74.9, 163 | **0.01** | 9.8, 33 | 6.9, 18 | 0.15 |
| Witnessed drug deals | 66.1, 177 | 71.7, 144 | 0.13 | 36.4, 80 | 24.3, 63 | **0.03** | 19.8, 44 | 17.7, 39 | 0.72 | 67.5, 188 | 53.5, 118 | **<0.001** | 1.3, 6 | 0.3, 1 | 0.30 |
| Witnessed gangs | 54.2, 133 | 45.7, 99 | 0.10 | 43.4, 108 | 14.5, 41 | **<0.001** | 32.1, 70 | 25.4, 59 | 0.34 | 76.7, 211 | 73.1, 153 | 0.30 | 5.0, 19 | 8.0, 19 | **0.02** |
| Witnessed someone being beaten up | 50.1, 144 | 51.6, 118 | 0.66 | 68.9, 169 | 60.1, 154 | 0.13 | 51.0, 107 | 48.0, 104 | 0.45 | 75.6, 219 | 76.8, 167 | 0.58 | 22.1, 62 | 17.2, 41 | 0.18 |
| Witness somebody pull a gun, knife etc. | 40.6, 103 | 28.7, 62 | 0.05 | 12.0, 34 | 13.0, 35 | 0.65 | 21.6, 42 | 18.0, 40 | 0.53 | 55.8, 155 | 37.0, 93 | **<0.001** | 2.9, 12 | 1.5, 4 | 0.14 |
| Witness someone get killed | 23.5, 74 | 27.2, 57 | 0.43 | 11.8, 30 | 18.0, 43 | 0.08 | 9.6, 16 | 9.5, 22 | 0.99 | 44.7, 106 | 28.4, 66 | **0.003** | 1.5, 6 | 0.6, 2 | **<0.001** |
| House broken into | 18.0, 57 | 18.5, 41 | 0.94 | 15.2, 44 | 21.2, 61 | 0.19 | 17.1, 37 | 14.9, 37 | 0.50 | 31.6, 78 | 25.3, 50 | 0.37 | 4.8, 19 | 2.8, 9 | 0.56 |

W% = weighted percentage; n = number of cases; N = sample size
