# Supplementary material for: Intersections between polyvictimisation and mental health among adolescents in five urban disadvantaged settings: the role of gender
Source: BMC Public Health. 2017 Jul 4;17(Suppl 3):41–50. doi: 10.1186/s12889-017-4348-y (PMC5498854; doi:10.1186/s12889-017-4348-y)
Supplement: Supplementary file 2 — Prevalence of peer violence, by gender within each city. (DOCX 32 kb) [file 12889_2017_4348_MOESM2_ESM.docx]

**Table S2: Prevalence of peer violence, by gender within each city**

|  | **Baltimore**  **W%, n** | | | **Delhi**  **W%, n** | | | **Ibadan**  **W%, n** | | | **Johannesburg**  **W%, n** | | | **Shanghai**  **W%, n** | | |
| --- | --- | --- | --- | --- | --- | --- | --- | --- | --- | --- | --- | --- | --- | --- | --- |
| **Category of violence** | **Male**  **N = 276** | **Female**  **N = 195** | ***P*** | **Male**  **N = 250** | **Female**  **N = 250** | ***P*** | **Male**  **N = 233** | **Female**  **N = 232** | ***P*** | **Male**  **N = 273** | **Female**  **N = 224** | ***P*** | **Male**  **N = 235** | **Female**  **N = 220** | ***P*** |
| **Peer violence** | 39.6, 104 | 37.2, 72 | 0.40 | 77.3, 179 | 46.9, 96 | **0.01** | 72.4, 59 | 47.5, 42 | 0.23 | 67.6, 130 | 63.9, 133 | 0.66 | 61.6, 40 | 44.3, 33 | **0.02** |
| Pushed, grabbed or shoved by someone | 30.0, 74 | 32.7, 56 | 0.60 | 62.5, 146 | 28.0, 61 | **0.001** | 37.5, 32 | 27.5, 24 | 0.47 | 39.2, 79 | 46.8, 96 | 0.20 | 55.0, 35 | 40.3, 28 | 0.19 |
| Hurt in fight | 13.6, 35 | 10.5, 24 | 0.28 | 34.9, 91 | 29.6, 64 | 0.06 | 31.0, 24 | 21.7, 19 | 0.52 | 30.9, 53 | 15.5, 34 | **0.02** | 19.0, 10 | 6.2, 6 | 0.13 |
| Threatened verbally | 19.6, 52 | 13.2, 30 | 0.06 | 38.0, 88 | 17.1, 31 | **<0.001** | 45.5, 37 | 23.4, 20 | 0.26 | 34.0, 72 | 37.2, 72 | 0.49 | 10.1, 10 | 10.9, 8 | 0.53 |
| Threatened with gun, knife or other weapon | 6.8, 25 | 4.4, 11 | 0.16 | 4.6, 13 | 6.3, 11 | 0.19 | 21.1, 12 | 12.9, 12 | 0.47 | 17.4, 38 | 8.4, 23 | **0.001** | 2.7, 3 | 0.9, 1 | **0.04** |
| Shot with gun | 3.3, 16 | 3.1, 8 | 0.68 | 6.8, 13 | 2.9, 6 | 0.14 | 10.2, 10 | 17.7, 16 | 0.09 | 11.8, 20 | 3.9, 10 | **<0.001** | 1.8, 3 | 2.3, 1 | 0.21 |

W% = weighted percentage; n = number of cases; N = sample size
