# Supplementary material for: Intersections between polyvictimisation and mental health among adolescents in five urban disadvantaged settings: the role of gender
Source: BMC Public Health. 2017 Jul 4;17(Suppl 3):41–50. doi: 10.1186/s12889-017-4348-y (PMC5498854; doi:10.1186/s12889-017-4348-y)
Supplement: Supplementary file 3 — Prevalence of intimate partner and non-partner sexual violence, by gender within each city. (DOCX 35 kb) [file 12889_2017_4348_MOESM3_ESM.docx]

**Table S3: Prevalence of intimate partner and non-partner sexual violence, by gender within each city**

|  | **Baltimore**  **W%, n** | | | **Delhi**  **W%, n** | | | **Ibadan**  **W%, n** | | | **Johannesburg**  **W%, n** | | | **Shanghai**  **W%, n** | | |
| --- | --- | --- | --- | --- | --- | --- | --- | --- | --- | --- | --- | --- | --- | --- | --- |
| **Category of violence** | **Male**  **N = 276** | **Female**  **N = 195** | ***P*** | **Male**  **N = 250** | **Female**  **N = 250** | ***P*** | **Male**  **N = 233** | **Female**  **N = 232** | ***P*** | **Male**  **N = 273** | **Female**  **N = 224** | ***P*** | **Male**  **N = 235** | **Female**  **N = 220** | ***P*** |
| **Physical IPV** | – | 25.1, 48 | – | – | 15.9, 7 | – | – | 25.6, 13 | – | – | 30.6, 65 | – | – | 7.9, 9 | – |
| Slap | – | 15.0, 24 | – | – | 5.3, 4 | – | – | 20.0, 9 | – | – | 24.5, 51 | – | – | 2.4, 5 | – |
| Push | – | 21.7, 39 | – | – | 12.8, 4 | – | – | 7.6, 5 | – | – | 19.0, 42 | – | – | 7.9, 9 | – |
| Punch | – | 14.1, 27 | – | – | 1.0, 1 | – | – | 9.3, 6 | – | – | 12.3, 31 | – | – | 1.0, 3 | – |
| Kick | – | 8.9, 17 | – | – | 0.0, 0 | – | – | 8.2, 5 | – | – | 10.1, 24 | – | – | 0.7, 2 | – |
| Strangle | – | 6.1, 13 | – | – | 2.1, 2 | – | – | 3.4, 2 | – | – | 2.4, 6 | – | – | 0.4, 1 | – |
| Knife | – | 7.4, 16 | – | – | 2.6, 2 | – | – | 4.4, 2 | – | – | 6.8, 13 | – | – | 0.7, 2 | – |
| **Sexual IPV** | – | 9.9, 19 | – | – | 7.4, 5 | – | – | 15.4, 11 | – | – | 19.5, 44 | – | – | 1.7, 4 | – |
| Forced sex | – | 5.7, 13 | – | – | 7.4, 5 | – | – | 10.3, 7 | – | – | 11.2, 22 | – | – | 1.3, 3 | – |
| Pressured on sex | – | 9.9, 19 | – | – | 6.3, 4 | – | – | 13.9, 10 | – | – | 15.1, 36 | – | – | 1.1, 3 | – |
| Threatened | – | 5.3, 12 | – | – | 4.6, 3 | – | – | 7.0, 5 | – | – | 10.2, 17 | – | – | 0.4, 1 | – |
| **Non-partner SV** | 15.1, 42 | 48.7, 112 | **<0.001** | 16.7, 42 | 33.4, 85 | **0.04** | 50.0, 97 | 26.7, 53 | **<0.001** | 34.1, 98 | 49.4, 117 | **0.006** | 16.4, 43 | 34.3, 66 | **0.001** |
| Harassment | 0.0, 0 | 42.6, 101 | **0.001** | 0.0, 0 | 31.2, 76 | **0.002** | 0.0, 0 | 12.1, 26 | **0.01** | 0.0, 0 | 39.2, 88 | **<0.001** | 0.0, 0 | 30.1, 58 | **0.001** |
| Unwanted sexual touching | 10.6, 34 | 13.5, 27 | 0.50 | 12.9, 32 | 5.7, 15 | **0.004** | 41.7, 82 | 18.7, 37 | **<0.001** | 25.7, 69 | 24.4, 54 | 0.66 | 13.9, 36 | 4.2, 12 | **0.001** |
| Coerced to have sex (pressure) | 11.5, 26 | 10.9, 21 | 0.89 | 8.4, 24 | 1.5, 6 | **0.001** | 24.4, 48 | 7.1, 14 | **<0.001** | 22.9, 59 | 9.3, 23 | **<0.001** | 5.0, 14 | 0.6, 2 | **<0.001** |
| Physical coercion or rape | 5.4, 19 | 10.2, 17 | 0.16 | 5.4, 14 | 0.8, 4 | **0.003** | 20.0, 43 | 4.8, 9 | **<0.001** | 15.6, 40 | 9.4, 25 | **0.03** | 2.1, 10 | 1.0, 2 | **0.04** |

W% = weighted percentage; n = number of cases; N = sample size
